# Long non‐coding RNA‐H19 stimulates osteogenic differentiation of bone marrow mesenchymal stem cells via the microRNA‐149/*SDF‐1* axis

**DOI:** 10.1111/jcmm.15040

**Published:** 2020-03-21

**Authors:** Guangjie Li, Xiangdong Yun, Kaishan Ye, Haiyan Zhao, Jiangdong An, Xueliang Zhang, Xingwen Han, Yanhong Li, Shuanke Wang

**Affiliations:** ^1^ Department of Orthopedics Lanzhou University Second Hospital Lanzhou China; ^2^ The First Hospital of Lanzhou University Lanzhou China

**Keywords:** bone defects, bone marrow mesenchymal stem cells, long non‐coding RNA‐H19, microRNA‐149, osteogenic differentiation, stromal cell‐derived factor‐1

## Abstract

Bone defects resulting from non‐union fractures or tumour resections are common clinical problems. Long non‐coding RNAs (lncRNAs) are reported to play vital roles in stem cell differentiation. The aim of this study was to elucidate the role of lncRNA‐H19 in osteogenic differentiation of bone marrow mesenchymal stem cells (BMMSCs). Following the establishment of an osteogenic differentiation model in rats, the expression of H19, microRNA‐149 (miR‐149) and stromal cell‐derived factor‐1 (SDF‐1) was measured by RT‐qPCR. Thereafter, BMMSCs were isolated from rats and treated with a series of mimic, inhibitor or siRNA. SDF‐1 expression, alkaline phosphatase (ALP) activity and osteocalcin (OCN) content were detected. The mineralized and calcified nodules were assessed by alizarin red S and Von Kossa staining. BMMSC surface markers were detected by flow cytometry. Western blot analysis was used to measure the expression of ALP, OCN, runt‐related transcription factor 2 (RUNX2) and osterix (OSX) proteins. Lastly, dual‐luciferase reporter gene assay and RNA immunoprecipitation were applied to verify the relationship of H19, miR‐149 and SDF‐1. Overexpressed H19 and SDF‐1 and poorly expressed miR‐149 were found in rats with osteogenic differentiation. H19 increased SDF‐1 expression by binding to miR‐149. H19 enhanced ALP activity, OCN content, calcium deposit and ALP, OCN, RUNX2 and OSX protein expression of BMMSCS by up‐regulating SDF‐1 via binding to miR‐149. Taken together, up‐regulated H19 could promote the osteogenic differentiation of BMMSCs by increasing SDF‐1 via miR‐149.

## 1 INTRODUCTION

The regeneration of large bone defects, whether congenital in nature or induced by trauma or diseases, remains clinically challenging.^1^ Bone possesses an inherent ability to regenerate, respond to injury as part of the repair process.^2^ However, delays in bone repair were linked to the increased joint stiffness, muscle atrophy, disuse bone loss and even elevated risk of falls.^3^ Differentiation of bone marrow mesenchymal stem cells (BMMSCs) during osteoblast formation is essential for the construction of bone.^4^ BMMSCs can differentiate into a variety of mature cell types, such as osteoblasts and adipocytes, under the influence of genetic and molecular mediators and local microenvironments.^5^ Although many experimental and clinical studies have tried to repair or regenerate bones with mesenchymal stem cells (MSCs), the comprehensive molecular mechanisms controlling osteogenic differentiation of MSCs have not been fully elucidated.^6^, ^7^ Thus, this calls for newer and more accurate predictors to provide a better prognosis of bone defect repair by mediating osteogenic differentiation of MSCs.

As a group of non–protein‐coding transcripts with nucleotides lengths of 200 or more, long non‐coding RNAs (lncRNAs) play pivotal roles in bone diseases.^8^ Several lncRNAs have been implicated in bone‐related disorders, including ANCR, ZBED3‐AS1 and H19.^9^, ^10^ Notably, H19 deficiency was found to inhibit tension‐induced osteogenic differentiation in human BMMSCs.^11^ A recent study has identified H19 as a microRNA (miRNA) sponge to reduce the endogenous function of miR‐141 and miR‐22 to actively modulate osteoblast differentiation.^12^ miR‐149 has been reported to be involved in regulating calcium ions, bone matrix mineralization and bone resorption, as well as differentiation and maintenance of bone tissue by targeting several pathways and genes.^13^ Furthermore, miR‐149 has been implicated in osteosarcoma as osteosarcoma progression was found enhanced by up‐regulated miR‐149 expression via the regulation of bone morphogenetic protein 9.^14^ A bioinformatics website microRNA.org revealed stromal cell‐derived factor‐1 (SDF‐1; also known as chemokine CXCL12) as a target gene of miR‐149. Notably, SDF‐1 has been shown to play vital roles in induction of cell recruitment, vascularization and osteogenic differentiation.^15^ In addition, AMD3100, an antagonist of the CXCR4/SDF‐1 interaction, has been found to markedly promote MSC mobilization efficiency in rats treated with the hypoxia‐mimicking agent CoCl_2_.^16^ Based on these findings, we hypothesized that he H19/miR‐149/SDF‐1 network might affect the osteogenic differentiation of BMMSCs.

## 2 MATERIALS AND METHODS

### 2.1 Ethical statement

All animal experiments in this study were in accordance with the principles of Guiding Opinions on the Treatment of Laboratory Animals by the Ministry of Science and Technology of the People's Republic of China. In addition, the study was conducted in strict accordance with the guidelines for the protection and use of laboratory animals published by the US National Institutes of Health, as well as the principle of completing experiments with a minimum number of animals and minimizing the suffering of all experimental animals.

### 2.2 Membrane‐induced osteogenic differentiation model establishment

Twenty‐four specific pathogen‐free (SPF) Sprague Dawley rats (aged 8 weeks and weighing 260‐280 g) were obtained from Better Biotechnology Co., Ltd. (No. J001), with 12 rats in the membrane‐induced group and 12 rats in the sham group. Then, rats were housed in cages at a temperature of 24‐26°C with humidity of 50%‐70%, controlled air flow and light (14‐hours day, 10‐hours night) and received water and food ad libitum. A rat model with membrane‐induced osteogenic differentiation was established as previously reported.^17^ The rats in the two groups were fasted for 24 hours with free access to water and then anaesthetized by intraperitoneal injection of 3% pentobarbital sodium (No. P3761, Sigma‐Aldrich Chemical Co). The rats were placed in the right lateral position, and 75% ethanol was used to disinfect the surgical area on the right leg. A longitudinal incision was made in the skin, and superficial fascia over the right femur, and the tensor fascia lata, biceps femoris and vastus lateralis muscles were bluntly separated from the greater trochanter to the femoral condyle exposing the lateral aspect of the femoral bone. A six‐hole stainless steel plate was applied to the centre of the lateral aspect of the femur shaft. After the plate was completely secured, the distal and proximal femur was completely truncated by repeating the above steps. The bone defect of rats in the membrane‐induced group was filled with polymethyl methacrylate cement, and the sham group was left untreated. The fascia was wounded and sutured in both groups. Following the irrigation with saline, the muscles and deep fascia were sutured with 4‐0 Vicryl sutures (Johnson and Johnson), and the skin was closed with 1‐0 sutures. Penicillin (penicillin sodium, North China pharmaceutical Co., Ltd, 1.6 million U/bottle, No. H13020655) was administered intramuscularly for 5 days to prevent infection. The rats were deprived of water and food for 12 hours and allowed full weight‐bearing activities with no restriction of movement. All rats were kept in a single cage for 4 days until the skin incision healed.

### 2.3 Isolation, culture and characterization of BMMSCs

SPF male rats (aged 8 weeks; Better Biotechnology Co., Ltd; No. J001) were soaked with 70% isopropanol, and the hindlimbs were clipped. Then, the central knee joints of the rats were dissected with sterile sharp scissors to remove ligaments and excess tissues. The femur and tibia were severed at the hip and ankle, respectively. After the surrounding muscles, ligaments and excess tissues were separated from the bone, the ends of the long bones were trimmed to expose the inside of the bone marrow. Subsequently, the femoral epiphyses were dissected, and the medulla was carefully washed with 3 mL low‐glucose Dulbecco's modified Eagle's medium containing 10% foetal bovine serum. The same needle and syringe were used to gently scratch the medulla several times on the ice to form a single cell suspension. The bone marrow suspension was then cultured in a polystyrene six‐well culture dish. After 2 days of culture, non‐adherent cells were washed away with phosphate buffered saline (PBS), and then, the medium was renewed. Adherent cells (P0) were cultured in a monolayer at 37°C with 5% CO_2_, and the medium was renewed every 3 days. Confluent cells were detached with 0.25% trypsin and 0.05% ethylenediaminetetraacetic acid (EDTA) and then passaged at a density of 5 × 10^4^ cells/well in a new 6‐well culture dish. After detachment, BMMSCs at P3 were seeded on 6‐well plates at a density of 2 × 10^4^/cm^2^. At 100% confluence, BMMSCs were cultured in growth medium, and the medium was renewed with adipose differentiation medium (Cyagen Biosciences Inc) for a 3‐day culture. Thereafter, the BMMSCs were cultured with adipose‐maintaining medium for 24 hours. After three cycles of adipose differentiation medium and replacement with adipose‐maintaining medium, the BMMSCs were cultured in adipose‐maintaining medium for another 7 days. After that, the BMMSCs were rinsed three times with PBS and stained with oil red O at room temperature for 10 minutes. The lipid droplets of BMMSCs were observed under a microscope. BMMSCs at P3 were seeded into 6‐well plates at a density of 3 × 10^4^/cm^2^. After 24 hours of culture in growth medium, BMMSCs were cultured in osteogenic differentiation medium (Cyagen Biosciences, Inc). The osteogenic differentiation medium was renewed every 3 days. After 2 weeks, BMMSCs were stained with alizarin red to observe osteogenic differentiation.

### 2.4 Cell transfection

Cell transfection was performed as previously reported.^18^ In brief, mimic‐NC, miR‐149 mimic, inhibitor‐NC, miR‐149 inhibitor, sh‐NC and sh‐H19 at final concentrations of 50 nM were transfected into BMMSCs using lipofectamine 3000 transfection reagent (Invitrogen) according to the manufacturer's protocol. After 48 hours of transfection, the BMMSCs were collected and used for further analysis.

### 2.5 Von Kossa staining and alizarin red S staining

The cell slides were fixed with acetone, rinsed with calcium‐ and magnesium‐free PBS (pH = 7.2), fixed at room temperature for 60 minutes with 70% ethanol and incubated with 5% silver nitrate at room temperature for 30 minutes under ultraviolet light. After distilled water washing, the slides were reacted with 5% sodium thiosulphate for 5 minutes and counterstained with neutral red for 20 minutes. Von Kossa staining of cells in each group was observed under a microscope to detect calcium deposition of cells in each group. In the images, calcium was stained in black and the nucleus in red.

After the removal of the inducer (10 nmoL/L dexamethasone, 10 mmoL/L β‐glycerophosphate and 50 μg/mL ascorbic acid), the cells were fixed with 1 mL 4% paraformaldehyde for 30 minutes at room temperature and stained with 1 mL 0.5% alizarin red at room temperature for 10 minutes. The BMMSCs were induced to differentiate, and 50 pg/mL vitamin C and 10 mmol/L β‐glycerol phosphate was added to the culture medium for culturing. After 21 days of continuous culture, the cells were stained with alizarin red S. Following the removal of the culture medium, the cells were fixed with 0.05% glutaraldehyde for 10 minutes, washed three times with deionized water and added with 0.4% alizarin red S. When the red staining accumulated, the staining solution was discarded, and the reaction was terminated with ddH_2_O. The staining was observed and imaged under a microscope.

### 2.6 RNA isolation and quantitation

Total RNA was isolated from the BMMSCs using Trizol reagent (R035102, Beijing Huamaike Biotechnology Co., Ltd). RNA was reversely transcribed into complementary DNA (cDNA) according to the instructions of a Primescript™ RT reagent kit (RR047A, Beijing Mairui Biotechnology Co., Ltd). The cDNA was used for quantitative polymerase chain reaction (qPCR) using a SYBR^®^ Premix Ex Taq™ II kit. Fluorescence quantitative PCR was performed on the ABI PRISM^®^ 7300 system using a reverse transcription‐quantitative PCR (RT‐qPCR) instrument (ViiA 7, Daan Gene Co., Ltd., of Sun Yat‐sen University). U6 and glyceraldehyde‐3‐phosphate dehydrogenase (GAPDH) served as loading controls for miR‐149 and other genes, respectively. The expression was calculated using relative quantification (2^−ΔΔCt^ method). The miRNA primer sequence was provided by the miRNA cDNA synthesis kit and the miRNA RT‐qPCR SYBR kit (Takara), and other primers were synthesized by Shanghai Sangon Biotechnology Co. Ltd (Table 1).

**Table 1:** Primer sequences for RT‐qPCR

Gene	Primer sequence
miR‐149	F: 5'‐GTTTGTGGCTCCGTGT‐3'
	R: 5'‐CAGTGCCTGTCGTGGAGT‐3'
lncRNA H19	F: 5'‐GATGGAGAGGACAGAAGGACAGT‐3'
	R: 5'‐GAGAGCAGCAGAGATGTGTTAGC‐3'
SDF‐1 (CXCL12)	F: 5'‐GGGAAACGGAGAAAGCTACC‐3'
	R: 5'‐CCCTCACCACACACACATCA‐3'
U6	F: 5'‐CTCGCTTCGGCAGCACA‐3'
	R: 5'‐AACGCTTCAGAATTTGCGT‐3'
GAPDH	F: 5'‐GCCAAGGTCATC‐3'
	R: 5'‐GAGGGGCCATCCACAGTCTT‐3'

Abbreviations: F, forward; GAPDH, glyceraldehyde‐3‐phosphate dehydrogenase; lncRNA H19, long non‐coding RNA H19; miR‐149, microRNA‐149; R, reverse; RT‐qPCR, reverse transcription‐quantitative polymerase chain reaction; SDF‐1, stromal cell‐derived factor‐1.

### 2.7 Determination of alkaline phosphatase (ALP) and osteocalcin (OCN)

BMMSCs were lysed by Triton X‐100, and protein was extracted by a 15‐minutes centrifugation at   10,656 *g*. ALP staining was performed according to instructions of the ALP kit (CD‐0664‐LIN, Wuhan Chundu Biotechnology Co., Ltd). In brief, protein was added with 50 μL ALP substrate reaction solution and colour developer. The absorbance at a wavelength of 405 nm (A405) was measured using a microplate reader (CLARIOstar, Bio‐Gene Technology Limited), and the ALP enzyme activity of BMMSCs was calculated. For measurement of OCN, lysed BMMSCs were centrifuged at   3108 *g* for 10 minutes, and 100 μL supernatant was harvested for detection. According to the instructions of the rat OCN enzyme‐linked immunosorbent analysis (ELISA) kit (MM‐0622R1, Jiangsu Jingmei Biotechnology Co., Ltd), the optical density (OD) value at a wavelength of 450 nm was detected using a microplate reader (CLARIOstar, Bio‐Gene Technology Limited), and OCN protein level was calculated.

### 2.8 Flow cytometry

BMMSCs were detached with 0.05% trypsin‐EDTA and washed two times with PBS. Cell suspensions of a density of 1 × 10^6^ cells/L were incubated with antibodies against CD29 phycoerythrin (PE; 562154, 1:100, 20 µL, BD Biosciences), CD44‐PE (ab23396, 1:200, 20 µL, Abcam), CD90‐PE (551401, 1:100, 20 µL, BD Biosciences) and CD45‐PE (554878, 1:100, 20 µL, BD Biosciences). The remaining cells served as the blank control through induction with PBS in the dark at 4°C for 30 minutes. After centrifugation, BMMSCs were washed two times with PBS. The expression of CD29, CD44, CD90 and CD45 was evaluated using a flow cytometer (FACScan; BD Biosciences). Data were analysed using the CellQuest software (BD Biosciences).

### 2.9 Dual‐luciferase reporter gene assay

Target gene analysis of miR‐149 was performed using a biological prediction website, and whether miR‐149 could bind to H19 and SDF‐1 was verified. Next, SDF‐1 3’ untranslated region (3’ UTR) fragment and H19 sequence were obtained by PCR amplification. The target segments were digested by the Xho I and Not I restriction endonuclease, cloned into the pmirGLO vector (E1330, Promega) on the downstream of luciferase reporter gene and transformed into *E coli* DH5a cells. The plasmids were amplified and named as wild‐type (SDF‐1‐WT and H19‐WT) and mutant type (SDF‐1‐MUT and H19‐MUT), respectively. The correctly sequenced luciferase reporter plasmids WT and MUT were cotransfected with miR‐149 mimic and mimic‐NC into HEK‐293T cells (Chinese Academy of Sciences Cell Bank; http://www.cellbank.org.cn/). After culture for 24 hours, the cells were lysed and centrifuged at 12 000 rpm for 4 minutes to collect the supernatant. The Dual‐Luciferase^®^ Reporter Assay System (RG006, Beyotime Institute of Biotechnology Co., Ltd) was applied to measure the luminescence activity.

### 2.10 RNA immunoprecipitation (RIP)

The binding of H19 and miR‐149 with argonaute 2 (AGO2) protein was verified according to the instructions of the RIP kit (Millipore Inc). A part of the cell extract was used as Input, and the other part was incubated with the antibodies and magnetic beads. The magnetic beads‐antibody complexes were resuspended in 900 μL RIP wash buffer. The samples were set on a magnetic base to collect the magnetic bead‐protein complexes. RNAs in the samples and input were separately extracted after detachment with protease K for the subsequent PCR detection. The antibodies for RIP included AGO2 (ab32381, 1:50, Abcam Inc) and IgG (1:100, ab109489, Abcam Inc) as the negative control.

### 2.11 Fluorescence in situ hybridization

Upon cell confluence of 60%‐70%, BMMSCs were fixed with 4% paraformaldehyde at room temperature for 10 minutes. BMMSCs were then permeabilized with 0.5% Triton X‐100 at 4°C for 15 minutes and incubated with red fluorescein‐labelled H19 probe (Guangzhou RiboBio Co., Ltd) at 37°C overnight. The cells were washed 6 times with pre‐heated 2× salt‐sodium citrate (each time for 3 minutes) and stained with 4′, 6‐diamidino‐2‐phenylindole. The slides were then sealed and photographed under a fluorescence microscope.

### 2.12 Western blot analysis

Total protein was extracted from BMMSCs according to the instructions of the Cellular Protein Extraction Kit (BC3640, Beijing Solarbio Science & Technology Co. Ltd). A bicinchoninic acid kit (20201ES76, Yisheng Biotechnology Co., Ltd) was used to determine protein concentration. Subsequently, cell lysates were resolved on 10% sodium dodecyl sulphate‐polyacrylamide gel electrophoresis and transferred onto a nitrocellulose membrane. After being blocked at 4°C overnight, the membrane was incubated with primary rabbit anti‐rat antibodies to SDF‐1 (ab25117, 1:1000), β‐actin (ab179467, 1:5000), ALP (ab194297, 1:1000), osterix (OSX, ab209484, 1:1000) or mouse anti‐rat antibodies to osteocalcin (ab13420, 1:1000), runt‐related transcription factor 2 (RUNX2; ab76956, 1:1000) overnight. Next, the membrane was incubated at 37°C for 1 hour with the following secondary antibodies, horseradish peroxidase‐labelled goat anti‐rabbit IgG (ab6721, 1:2000) or rabbit antimouse IgG (ab6728, 1:2000). All above‐mentioned antibodies were purchased from Abcam Inc. Immune complexes were developed using enhanced chemiluminescence (Pierce Biotechnology) at room temperature for 1 minute and visualized using an Image Quant LAS 4000C gel imager (GE company). β‐actin was used as the loading control. Protein expression was calculated as the ratio of the grey value of the target band to that of the loading control band, considered as the relative protein expression.

### 2.13 Statistical analysis

All data analysis was performed using the SPSS 21.0 statistical software (IBM Corp). Measurement data were presented as mean ± standard deviation. When data conformed to normal distribution and homogeneity of variance, comparisons between two groups were conducted using unpaired *t* test. The one‐way analysis of variance (ANOVA) with Tukey's post hoc test was used for comparisons among multiple groups. Correlation coefficients were calculated by Pearson's correlation test. *P* < .05 was considered to be statistically significant.

## 3 RESULTS

### 3.1 Identification of BMMSCs

**Figure 1:**
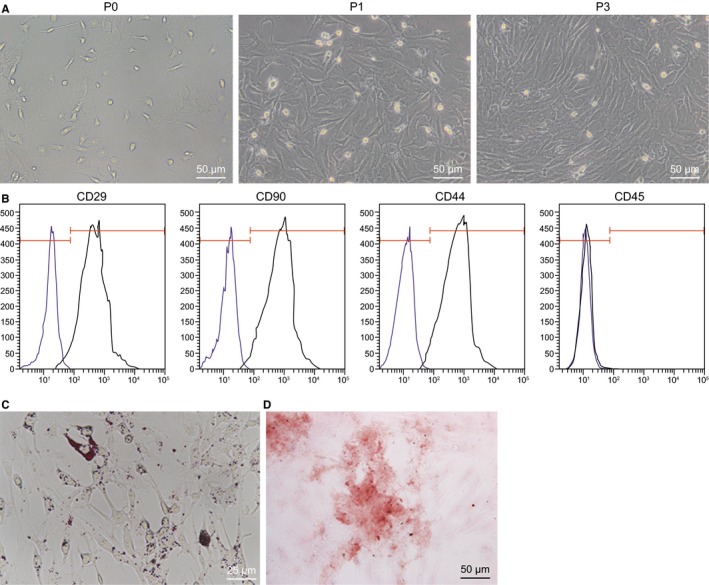
BMMSCs of rats are successfully isolated and cultured. A, Morphology of BMMSCs at P0, P1 and P3 observed under an inverted fluorescence microscopy (200×). B, Flow cytometry detection of the surface markers of BMMSCs (CD29, CD44, CD90 and CD45). C, adipogenic differentiation of BMMSCs was visualized by Oil red O staining (400×). D, Osteogenic differentiation of BMMSCs was visualized by alizarin red S (200×)

Initially, in order to verify that the isolated cultured cells were BMMSCs, the morphology of the cells was observed under an inverted fluorescence microscopy (Figure 1A). Thereafter, the surface markers (CD29, CD44, CD90 and CD45) in BMMSCs at the P3 were detected using flow cytometry. The results showed that CD29, CD44 and CD90 were overexpressed, which were 85.9%, 80.6% and 94.6%, while CD45 (0.046%) was poorly expressed (Figure 1B). These characteristics were similar to what was previously described for BMMSCs. In order to verify the adipogenesis and osteogenesis of BMMSCs, the cells were induced to differentiate in osteogenic and adipogenic media. As depicted in Figure 1C, 1, after adipogenic induction, cell proliferation slowed down with elliptical or polygonal cell morphology; meanwhile, the lipid droplets in the cytoplasm accumulated and were stained red by oil red O. After 3 weeks of osteogenic induction, the cell morphology changed from spindle to polygon and cells were seen arranged densely. Red calcium salt nodules were observed by Von Kossa staining.

### 3.2 H19 promotes osteogenic differentiation of BMMSCs

**Figure 2:**
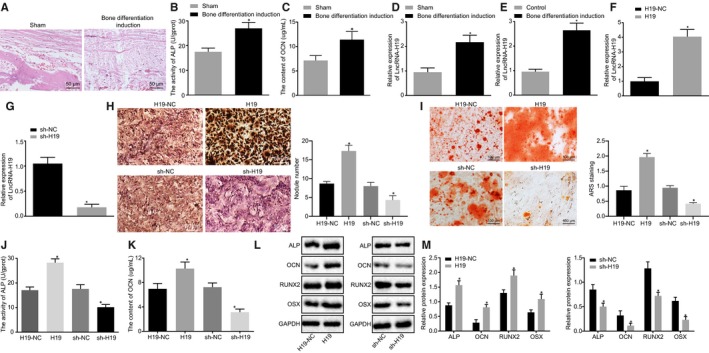
Overexpressed H19 is found in osteogenic differentiation. A, Von Kossa staining of calcium deposition in the periosteum of rats (200×). B, ALP activity in the periosteum of rats. C, OCN content in the periosteum of rats. D, H19 expression in the periosteum of rats. E, H19 expression in BMMSCs after osteogenic differentiation detected by RT‐qPCR. F, RT‐qPCR detection of H19 expression in BMMSCs after overexpression of H19. G, RT‐qPCR detection of H19 expression in BMMSCs after silencing of H19. H, The number of calcified nodules after silencing or overexpression of H19 measured by Von Kossa staining. I, The number of mineralized nodules after silencing or overexpression of H19 determined by alizarin red S. J, ALP activity after silencing or overexpression of H19. K, OCN content after silencing or overexpression of H19. L‐M, The protein expression of ALP, OCN, RUNX2 and OSX after silencing or overexpression of H19 measured by Western blot analysis. Abbreviations: ALP, alkaline phosphatase; OCN, osteocalcin; RUNX2, Runt‐related transcription factor 2; OSX, osterix. ^*^ *P* < .05 compared with the sham‐operated rats or BMMSCs without osteogenic differentiation. The results were measurement data, which were expressed as mean ± standard deviation. Comparisons between two groups were conducted by means of unpaired *t* test. The experiment was independently repeated three times. (n = 12)

A rat model with osteogenic differentiation was established by membrane induction technology. Von Kossa staining demonstrated that compared with the sham‐operated rats, severe calcium deposition was found in rats with osteogenic differentiation (Figure 2A). Next, ALP activity and OCN content in the periosteum were measured, and results showed that as compared with the sham‐operated rats, the ALP activity and OCN content were increased in rats with osteogenic differentiation (*P* < .05; Figure 2B,C).

RT‐qPCR was performed to examine the expression of H19 in the sham‐operated, and rats with osteogenic differentiation with the results showed that compared with the sham‐operated rats, the expression of H19 was notably increased in rats with osteogenic differentiation (*P* < .05; Figure 2D). Besides, the cells with osteogenic differentiation showed increased expression of H19 as compared to cells without osteogenic differentiation (*P* < .05; Figure 2E).

In order to verify whether H19 promotes osteogenic differentiation of BMMSCs, we constructed overexpression plasmids of H19 and shRNA plasmids to increase or decrease the expression of H19 in BMMSCs, respectively. The results of RT‐qPCR showed that H19 expression was significantly elevated in cells treated with overexpressed H19 (Figure 2F), but decreased in cells treated with sh‐H19 (Figure 2G). The number of calcified nodules, mineralized nodules and ALP activity in each group were observed by Von Kossa, alizarin red S (ARS) and ALP, respectively. The results showed that sh‐H19 contributed to lowered number of calcified and mineralized nodules and decreased ALP activity relative to sh‐NC. By contrast, the number of calcified and mineralized nodules and ALP activity following H19 treatment was significantly increased vs H19‐NC treatment (Figure 2H‐J). Western blot analysis showed that the expression of ALP, OCN, RUNX2 and OSX was significantly induced by H19 up‐regulation but reduced by treatment with sh‐H19 (Figure 2K‐M). These results together showed that H19 could promote osteogenic differentiation of BMMSCs.

### 3.3 H19 competitively binds to miR‐149

**Figure 3:**
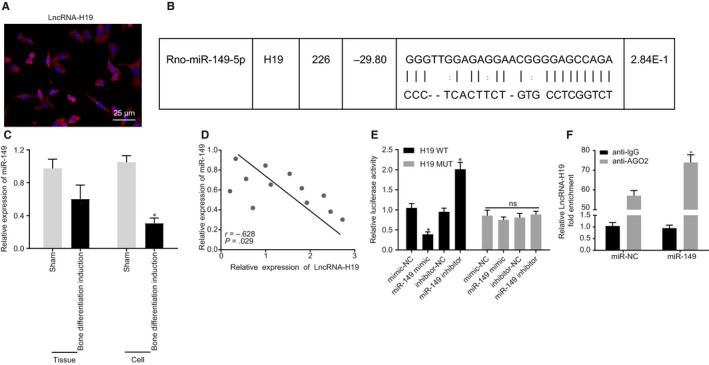
H19 binding to miR‐149 is predicted and verified. A, The subcellular localization of H19 using fluorescence in situ hybridization (400×). B, Schematic of miR‐149 and the putative binding sequence at the 3'UTR of H19. C, miR‐149 expression in rat models and BMMSCs. D, Pearson's correlation analysis of the correlation between miR‐149 and H19. E, Targeting relationship between miR‐149 and H19 evaluated by dual‐luciferase reporter gene assay. F, RIP detection of the binding relationship between miR‐149 and H19. ^*^ *P* < .05 compared with the sham‐operated rats, BMMSCs without osteogenic differentiation, mimic‐NC or inhibitor‐NC treatments. The results were measurement data, which were expressed as mean ± standard deviation. Comparisons between two groups were conducted by means of unpaired *t* test. Pearson correlation analysis was performed to assess the correlation between miR‐149 and H19. The cell experiment was independently repeated three times

Fluorescence in situ hybridization was used to detect the subcellular location of H19. The results showed that H19 was located in the cytoplasm and nucleus (Figure 3A). Bioinformatics prediction revealed that miR‐149 could bind to H19 (Figure 3B). RT‐qPCR was used to detect miR‐149 expression in rat models and BMMSCs. The results showed that miR‐149 expression was significantly decreased in BMMSCs and rats with osteogenic differentiation (Figure 3C). Pearson correlation analysis of rat model with osteogenic differentiation showed that there was a significant negative correlation between miR‐149 and H19 (*P* < .05; Figure 3D). In order to further confirm the binding of miR‐149 and H19, a dual‐luciferase reporter gene assay was performed. Luminescence activity in H19 WT was significantly diminished by miR‐149 mimic (*P* < .05), while that in H19 MUT was not affected (*P* > .05; Figure 3E). RIP results further confirmed the binding relationship between miR‐149 and H19 (Figure 3F). Overall, these findings demonstrated that H19 could bind to miR‐149.

### 3.4 H19 promotes osteogenic differentiation of BMMSCs by down‐regulating miR‐149

**Figure 4:**
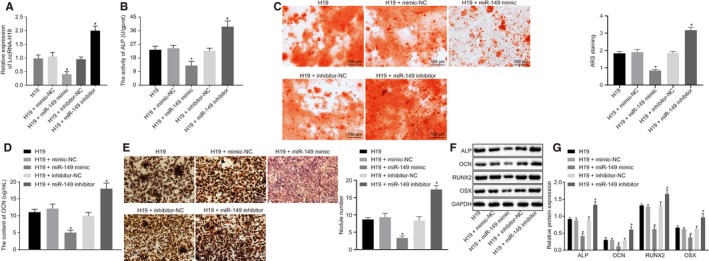
H19 induces osteogenic differentiation of BMMSCs via the decrease of miR‐149. BMMSCs with osteogenic differentiation were treated with H19, H19 + mimic‐NC, H19 + miR‐149 mimic, H19 + inhibitor‐NC and H19 + miR‐149 inhibitor. A, H19 expression in BMMSCs determined by RT‐qPCR. B, ALP activity in BMMSCs. C, The number of mineralized nodules determined by alizarin red S. D, OCN content in BMMSCs. E, Von Kossa staining of calcified nodules in BMMSCs. F‐G, The protein expression of ALP, OCN, RUNX2 and OSX after silencing or overexpression of miR‐149, in BMMSCs, in the presence of H19 measured by Western blot analysis. The band intensity was assessed. Abbreviations: ALP, alkaline phosphatase; OCN, osteocalcin; RUNX2, Runt‐related transcription factor 2; OSX, osterix. ^*^ *P* < .05 compared with BMMSCs treated with H19 + mimic‐NC or H19 + inhibitor‐NC. The results were measurement data, which were expressed as mean ± standard deviation. Comparisons between multiple groups were analysed by one‐way ANOVA with Tukey's post hoc test. The experiment was independently repeated three times

Next, in order to investigate the effects of H19 on osteogenic differentiation with the involvement of miR‐149, BMMSCs with osteogenic differentiation were treated with H19, H19+ mimic‐NC, H19+ miR‐149 mimic, H19+ inhibitor‐NC or H19+ miR‐149 inhibitor. Firstly, we showed that compared with treatment with H19+ mimic‐NC, H19 expression (Figure 4A), ALP activity (Figure 4B), the mineralized nodules (Figure 4C), OCN content (Figure 4D) and calcified nodules (Figure 4E) were diminished in treatment with H19+ miR‐149 mimic. However, the opposite findings were observed upon treatment with H19+ miR‐149 inhibitor in contrast to treatment with H19+ inhibitor‐NC (*P* < .05). The results of Western blot analysis showed that, compared with the corresponding controls, ALP, OCN, RUNX2 and OSX expression was significantly down‐regulated in BMMSCs treated with H19+ miR‐149 mimic but increased in BMMSCs treated with H19+ miR‐149 inhibitor (Figure 4F‐4G). Together, these findings revealed H19 could enhance osteogenic differentiation of BMMSCs by down‐regulating miR‐149.

### 3.5 miR‐149 represses osteogenic differentiation of BMMSCs

**Figure 5:**
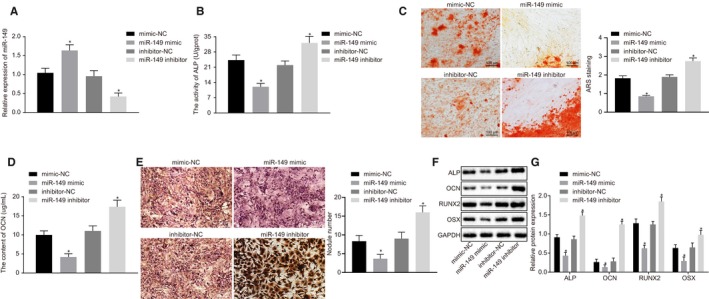
Overexpressed miR‐149 inhibits osteogenic differentiation of BMMSCs. BMMSCs with osteogenic differentiation were treated with mimic‐NC, miR‐149 mimic, inhibitor‐NC and miR‐149 inhibitor. A, miR‐149 expression in BMMSCs determined by RT‐qPCR. B, ALP activity in BMMSCs. C, The number of mineralized nodules determined by alizarin red S. D, OCN content in BMMSCs. E, Calcified nodules in BMMSCs determined by Von Kossa staining. F‐G, Western blot analysis of ALP, OCN, RUNX2 and OSX expression in BMMSCs. The band intensity was assessed. Abbreviations: ALP, alkaline phosphatase; OCN, osteocalcin; RUNX2, Runt‐related transcription factor 2; OSX, osterix. ^*^ *P* < .05 compared with BMMSCs treated with mimic‐NC or inhibitor‐NC. The results were measurement data, which were expressed as mean ± standard deviation. Comparisons between two groups were conducted by means of unpaired t test. The experiment was independently repeated three times

The expression of miR‐149 was altered in BMMSCs with osteogenic differentiation to further verify whether miR‐149 mediates osteogenic differentiation of BMMSCs. RT‐qPCR showed that the expression of H19 was notably increased in BMMSCs treated with miR‐149 mimic and decreased after treatment of miR‐149 inhibitor (Figure 5A). Furthermore, ALP activity (Figure 5B), mineralized nodules (Figure 5C), OCN content (Figure 5D) and calcified nodules (Figure 5E) were progressively diminished in BMMSCs treated with miR‐149 mimic, but increased in BMMSCs treated with miR‐149 inhibitor. Western blot analysis showed ALP, OCN, RUNX2 and OSX expression was significantly decreased in BMMSCs treated with miR‐149 mimic, but induced in BMMSCs treated with miR‐149 inhibitor (Figure 5F, G). Taken together, these data suggested that overexpressed miR‐149 could inhibit the osteogenic differentiation of BMMSCs.

### 3.6 miR‐149 targets and negatively regulates SDF‐1

**Figure 6:**
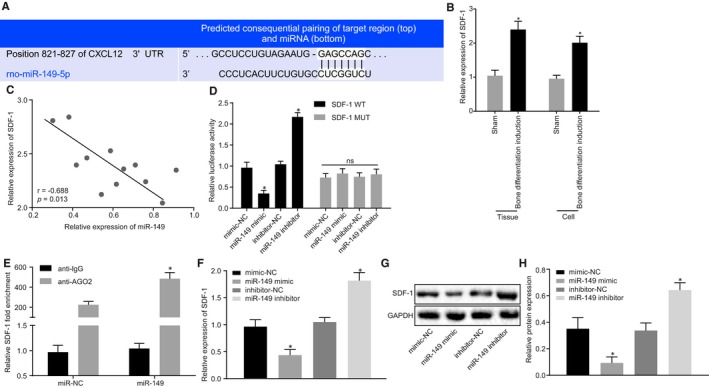
*SDF‐1* is verified as a target gene of miR‐149. A, Bioinformatics prediction of binding site of miR‐149 on the 3'UTR of *SDF‐1*. B, *SDF‐1* expression in rat models and BMMSCs with osteogenic differentiation. C, Pearson analysis of the correlation between miR‐149 and *SDF‐1*. D, Targeting relationship between miR‐149 and *SDF‐1* evaluated by dual‐luciferase reporter gene assay. E, RIP detection of the binding relationship between miR‐149 and *SDF‐1*. F, *SDF‐1* mRNA expression in BMMSCs after alteration of miR‐149. G‐H, Western blot analysis of SDF‐1 protein expression in BMMSCs after alteration of miR‐149. The band intensity was assessed. ^*^ *P* < .05 compared with sham‐operated rats, BMMSCs without osteogenic differentiation, BMMSCs treated with mimic‐NC or inhibitor‐NC or miR‐NC treatment. The results were measurement data, which were expressed as mean ± standard deviation. Comparisons between two groups were conducted by means of unpaired t test. Pearson correlation analysis was performed to assess the correlation between miR‐149 and *SDF‐1*. The experiment was independently repeated three times

Bioinformatics prediction indicated that miR‐149 could bind to SDF‐1 (Figure 6A). Thereafter, RT‐qPCR was conducted to determine the mRNA expression of SDF‐1 in BMMSCs with osteogenic differentiation. The results showed that SDF‐1 expression was significantly elevated in rats and BMMSCs with osteogenic differentiation (*P* < .05; Figure 6B). Pearson correlation analysis of rat model showed that there was a significant negative correlation between miR‐149 and SDF‐1 (*P* < .05; Figure 6C). In order to verify the targeting relationship between miR‐149 and SDF‐1, a dual‐luciferase reporter gene assay was performed. The results showed that the luminescence activity in SDF‐1 WT was notably reduced by miR‐149 mimic (*P* < .05), but that of SDF‐1 MUT was not affected (*P* > .05; Figure 6D). RIP assay further confirmed that miR‐149 bound to SDF‐1 (Figure 6E). To test whether miR‐149 affects SDF‐1 expression, RT‐qPCR and Western blot analysis were performed. The results showed that BMMSCs overexpressing miR‐149 showed down‐regulated SDF‐1 expression, whereas BMMSCs treated with miR‐149 inhibitor had elevated SDF‐1 expression (Figure 6F‐H). These results suggested that SDF‐1 expression could be promoted by down‐regulating miR‐149.

### 3.7 Up‐regulated H19 stimulates osteogenic differentiation of BMMSCs via up‐regulating SDF‐1 by binding to miR‐149

**Figure 7:**
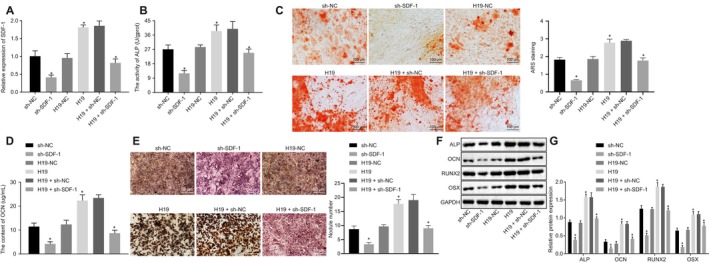
H19 elevates *SDF‐1* expression by binding to miR‐149 to induce osteogenic differentiation of BMMSCs. BMMSCs with osteogenic differentiation were introduced with sh‐NC, sh‐SDF‐1, H19‐NC, H19, H19 + sh‐NC and H19 + sh‐SDF‐1. A, mRNA expression of SDF‐1 in BMMSCs measured by RT‐qPCR. B, ALP activity in BMMSCs. C, The number of mineralized nodules determined by alizarin red S. D, OCN content in BMMSCs. E, Calcified nodules in BMMSCs determined by Von Kossa staining. F‐G, Western blot analysis of ALP, OCN, RUNX2 and OSX expression in BMMSCs. The band intensity was assessed. Abbreviations: ALP, alkaline phosphatase; OCN, osteocalcin; RUNX2, Runt‐related transcription factor 2; OSX, osterix. ^*^ *P* < .05 compared with BMMSCs treated with sh‐NC, H19‐NC or H19 + sh‐NC. The results were measurement data, which were expressed as mean ± standard deviation. Comparisons between two groups were conducted by means of unpaired t test. The experiment was independently repeated three times

Lastly, to further examine the relationship among H19, miR‐149 and SDF‐1, BMMSCs were introduced with sh‐NC, sh‐SDF‐1, H19‐NC, H19, H19+ sh‐NC and H19+ sh‐SDF‐1. Then, the effect of H19 and SDF‐1 on osteogenic differentiation was evaluated. As described in Figure 7A‐F, SDF‐1 expression, ALP activity, mineralized nodules, OCN content, calcified nodules and ALP, OCN, RUNX2 and OSX expression were notably reduced in BMMSCs following SDF‐1 silencing. Meanwhile, BMMSCs stimulated with H19 had increased SDF‐1 expression (Figure 7A), ALP activity (Figure 7B), mineralized nodules (Figure 7C), OCN content (Figure 7D) and calcified nodules (Figure 7E), as well as the protein expression of ALP, OCN, RUNX2 and OSX (Figure 7F, G), which was rescued by sh‐SDF‐1. These results suggested that H19 increased SDF‐1 expression by binding to miR‐149 and promoted the osteogenic differentiation of BMMSCs.

## 4 DISCUSSION

Increasing incidence of bone disorders along with ageing populations call for more effective therapies for bone regeneration.^19^ LncRNAs are known to affect osteoblast differentiation via a variety of regulatory mechanisms, such as modification of chromatin, binding to transcription factors and competing endogenous mechanisms, as well as other post‐transcriptional mechanisms.^20^ For fully clarifying the comprehensive molecular mechanisms mediating the osteogenic differentiation of BMMSCs, we studied the effects of H19 on the osteogenic differentiation of BMMSCs by miR‐149‐mediated regulation of SDF‐1. In our experiment, membrane‐induced osteogenic differentiation rat models were successfully established, and the collective results demonstrated that up‐regulated H19 could induce the expression of SDF‐1 through the inhibition of miR‐149, thereby promoting the differentiation of BMMSCs.

Our initial findings demonstrated that highly expressed H19 and poorly expressed miR‐149 were associated with osteogenic differentiation of BMMSCs. A prior study demonstrated elevated H19 expression in the cartilage differentiation of adipose‐derived stem cells into chondrocytes.^21^ Similar to our study, H19 expression was elevated after osteoblasts were induced to differentiate and increase of H19 stimulated human MSC osteogenic differentiation.^22^ In addition, up‐regulated miR‐149 was found in patients with osteonecrosis of the jaw, indicating that inhibited miR‐149 contributed to calcium ions, bone matrix mineralization, bone resorption and differentiation and maintenance of bone tissue.^13^ Further, lncRNAs, acting in tandem with miRs, have been found to post‐transcriptionally regulate many genes and simultaneously exerted many traits through numerous different targets.^23^ In our subsequent experiments, we demonstrated that H19 bound to miR‐149 and induced the osteogenic differentiation of BMMSCs. Consistently, Liang et al also demonstrated that H19 promoted osteoblast differentiation by serving as a miRNA sponge to diminish the endogenous functions of miR‐141 and miR‐22.^12^ Therefore, the overexpression of H19 and inhibition of miR‐149 exerted their therapeutic potential in bone defect repair by promoting osteogenic differentiation.

In our study, the RNA crosstalk between miR‐149 and SDF‐1 during osteogenic differentiation of BMMSCs was also investigated. We proved that SDF‐1 was a target gene of miR‐149 and up‐regulation of miR‐149 could down‐regulate SDF‐1 expression. In a similar finding, overexpressed miR‐149 was reported to target and inhibit fibroblast growth factor‐21 gene.^24^ Furthermore, induced SDF‐1 has been noted to contribute to enhanced bone formation and regeneration by stimulating cell recruitment, vascularization and osteogenic differentiation.^25^ In addition, SDF‐1 was related to promoted osteogenic differentiation of MSCs by working with related signalling pathways.^26^

Most importantly, we observed that H19 up‐regulated SDF‐1 by binding to miR‐149 and promoted osteogenic differentiation of BMMSCs, as supported by increased contents of ALP, OCN, RUNX2 and OSX. As the osteogenic differentiation markers, induced ALP, OCN and calcium deposition was directly associated with enhanced osteogenic differentiation.^27^ In a previous study, induced OCN was demonstrated to promote bone regeneration in cultured osteogenic MC3T3‐E1 cells.^28^ In addition, BMMSCs displayed significantly lower ALP activity and fewer mineralized nodules, as well as down‐regulated RUNX2, OXS and OCN after post‐menopausal osteoporosis.^29^ Further, angelica polysaccharide significantly enhanced cell viability and increased RUNX2, OCN and ALP in MSCs, which was reversed by H19 knockdown.^30^ Additionally, acceleration of osteogenic differentiation was achieved through the induction of exogenous CXCL12 (SDF‐1) in mesenchymal progenitor stem cells by elevating the markers of osteogenic differentiation (RUNX2, OCN and ALP).^27^ Moreover, H19 was found to promote the osteogenesis of human BMMSCs through binding to miR‐138 as well as inducing downstream focal adhesion kinase.^11^ Collectively, our results demonstrated that the lncRNA‐H19/miR‐149/SDF‐1 axis might be an important target for promoting osteogenic differentiation of BMMSCs.

## 5 CONCLUSIONS

**Figure 8:**
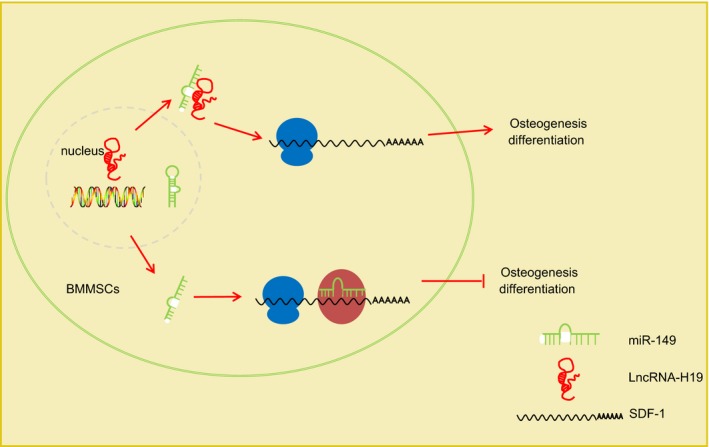
Mechanism of H19 in osteogenic differentiation of BMMSCs with the involvement of miR‐149 and *SDF‐1*. In BMMSCs, miR‐149 targets *SDF‐1* to inhibit *SDF‐1* expression, thus inhibiting osteogenic differentiation. When H19 exists, it can interact with miR‐149 to reduce the inhibitory effect of miR‐149 on *SDF‐1*, which promotes *SDF‐1* expression and then induces osteogenic differentiation

In summary, these data demonstrated that up‐regulated H19 could improve osteogenic differentiation of BMMSCs by binding to miR‐149 to up‐regulate SDF‐1 (Figure 8). Besides, in vivo and clinical experiments would be beneficial to prove the function of lncRNA‐H19/miR‐149/SDF‐1 axis in bone formation/regeneration. Although our findings provide further rational basics for the development of bone defect repair treatment, the specific mechanisms mediating the interactions between of H19, miR‐149 and other‐related signalling pathways still demand further elucidation.

## CONFLICT OF INTEREST

None.

## AUTHOR CONTRIBUTIONS

Guangjie Li and Yanhong Li designed the study. Xueliang Zhang collated the data. Kaishan Ye and Jiangdong An analyses and produced the initial draft of the manuscript. Xingwen Han, Shuanke Wang and Guangjie Li contributed to drafting the manuscript. Xiangdong Yun and Haiyan Zhao revised it critically for important intellectual content. All authors have read and approved the final submitted manuscript.

## Data Availability

Research data no shared.
