# Corrigendum

**DOI:** 10.1111/jcmm.17068

**Published:** 2022-01-08

In Guangjie Li, et al.,^1^ the H19 + mimic‐NC image in Figure 4C contains error. The correct figure is shown below. The authors confirm all results, conclusions of this article remain unchanged.

**FIGURE 4:**
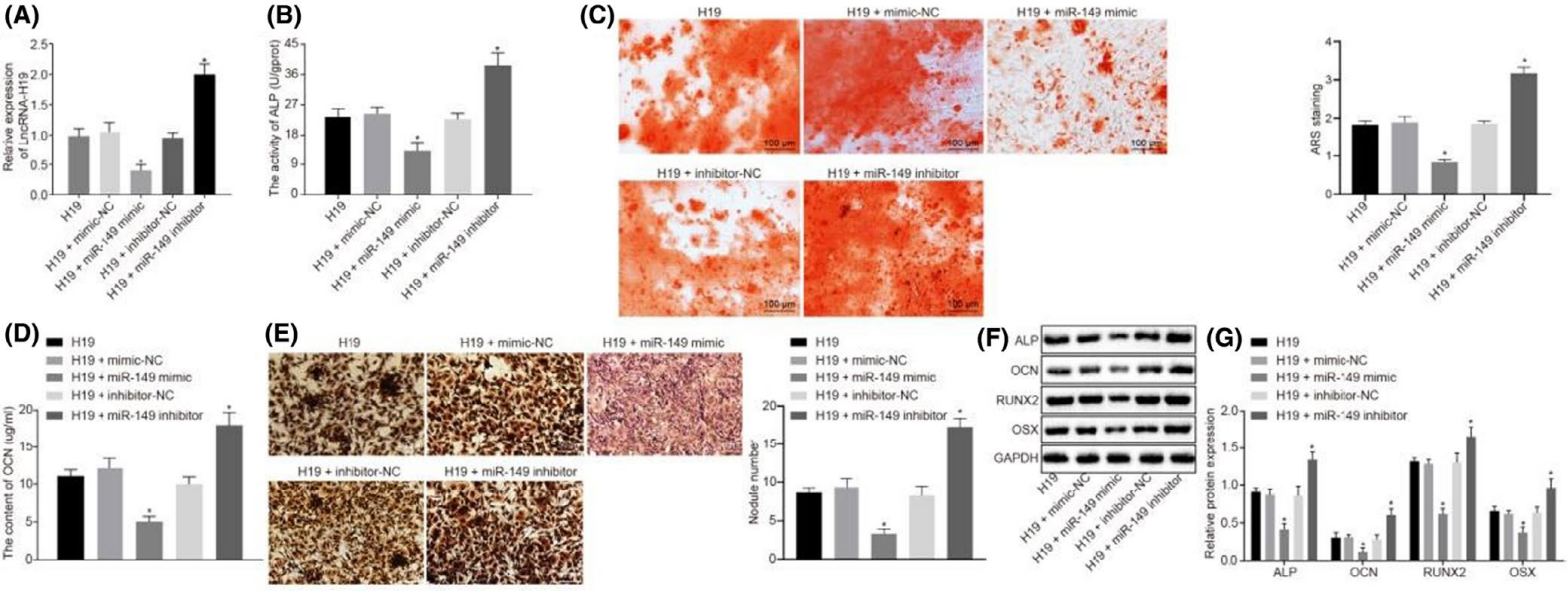
H19 induces osteogenic differentiation of BMMSCs via the decrease of miR‐149. BMMSCs with osteogenic differentiation were treated with H19, H19 + mimic‐NC, H19 + miR‐149 mimic, H19 + inhibitor‐NC and H19 + miR‐149 inhibitor. (A) H19 expression in BMMSCs determined by RT‐qPCR. (B) ALP activity in BMMSCs. (C) The number of mineralized nodules determined by alizarin red S. D, OCN content in BMMSCs. (E) Von Kossa staining of calcified nodules in BMMSCs. (F‐G) The protein expression of ALP, OCN, RUNX2 and OSX after silencing or overexpression of miR‐149, in BMMSCs, in the presence of H19 measured by Western blot analysis. The band intensity was assessed. Abbreviations: ALP, alkaline phosphatase; OCN, osteocalcin; RUNX2, Runt‐related transcription factor 2; OSX, osterix. ^*^ *p* < .05 compared with BMMSCs treated with H19 + mimic‐NC or H19 + inhibitor‐NC. The results were measurement data, which were expressed as mean ± standard deviation. Comparisons between multiple groups were analysed by one‐way ANOVA with Tukey's post hoc test. The experiment was independently repeated three times
