# Expression of Concern: Long Non‐Coding RNA‐H19 Stimulates Osteogenic Differentiation of Bone Marrow Mesenchymal Stem Cells via the MicroRNA‐149/SDF‐1 Axis

**DOI:** 10.1111/jcmm.70242

**Published:** 2024-12-12

EXPRESSION OF CONCERN: G. Li, X. Yun, K. Ye, H. Zhao, J. An, X. Zhang, X. Han, Y. Li, and S. Wang, “Long Non‐Coding RNA‐H19 Stimulates Osteogenic Differentiation of Bone Marrow Mesenchymal Stem Cells via the MicroRNA‐149/SDF‐1 Axis,” *Journal of Cellular and Molecular Medicine* 24, no. 9 (2020): 4944–4955. https://doi.org/10.1111/jcmm.15040.

This Expression of Concern is for the above article, published online on 21 March 2020 in Wiley Online Library (wileyonlinelibrary.com), and has been published by agreement between the journal Editor‐in‐Chief, Stefan Constantinescu; The Foundation for Cellular and Molecular Medicine; and John Wiley and Sons Ltd. A third party contacted the journal to express concerns regarding evidence of errors in the regression lines for Figures 3D and 6B. The third party also notified the journal of apparent errors in the flow cytometry graphs in Figure 1C. The authors did not respond to multiple inquiries by the publisher and requests to share the original data. The expression of concern has been agreed to because the journal is unable to determine the veracity of the data included in this article. The authors did not respond to our notice regarding the expression of concern.

